# Supplementary material for: Risk factors for healthcare-associated infections in children undergoing ECMO after cardiac surgery for congenital heart disease: A retrospective study
Source: PLoS One. 2026 May 11;21(5):e0348220. doi: 10.1371/journal.pone.0348220 (PMC13160298; doi:10.1371/journal.pone.0348220)
Supplement: S1 File — (PDF) [file pone.0348220.s001.pdf]

| gender | The location of ECMO initiation (operating room is 1/ICU is 2) | CPB time (min) | ascending aorta cross-clamping time (min) | admission diagnosis | Cannulation approach (central is 1vs. peripheral is 0) | age (Neonates record 1 for "Yes" and 0 for "No") | body weight [kg] | Blood lactate (mmol/l) | BUN (mmol/L) | serum creatinine (umol/L) | bilirubin (umol/L) | Albumin(g/L) | the blood transfusion volume 24 hours after ECMO (ml) | postoperative chest drainage volume within 24 hours[ml | ECPR (record 1 for "Yes" and 0 for "No") | peritoneal dialysis (record 1 for "Yes" and 0 for "No") | CRRT (record 1 for "Yes" and 0 for "No") | duration of ventilator support before ECMO (d) | preoperative en | the duration of ECMO assistance (d) | the length of stay in the ICU (d) | Pulmonary Infectio (record 1 for "Yes" and 0 for "No") | Bloodstream Infectio (record 1 for "Yes" and 0 for "No") | Urinary Tract Infectio (record 1 for "Yes" and 0 for "No") | Surgical Site Infectio (record 1 for "Yes" and 0 for "No") | Pathogen | Death (record 0 for "Yes" and 1 |
|---|---|---|---|---|---|---|---|---|---|---|---|---|---|---|---|---|---|---|---|---|---|---|---|---|---|---|---|
| female | 1 | 227 | 69 | Ventricular Septa | 1 | 1 | 2.7 | 6.53 | 11.5 | 72 | 26.5 | 37.2 | 100 | 135 | 0 | 1 | 1 | 0 | 0 | 2 | 2 | 0 | 0 | 0 | 0 | 0 | 0 |
| female | 1 | 258 | 94 | Truncus Arteriosu | 0 | 0 | 46.7 | 5.64 | 8.8 | 67 | 48.4 | 33 | 1590 | 640 | 0 | 0 | 1 | 0 | 0 | 10 | 23 | 0 | 0 | 0 | 0 | 0 | 1 |
| female | 2 | 164 | 124 | Transposition of the Great Arteries | 1 | 0 | 7.1 | 19.93 | 8.1 | 74 | 30.4 | 24.3 | 560 | 60 | 1 | 1 | 1 | 0 | 0 | 4 | 4 | 0 | 0 | 0 | 0 | 0 | 0 |
| female | 2 | 107 | 44 | Supravalvular Aor | 0 | 0 | 27.5 | 3.7 | 10.4 | 72 | 49.1 | 49 | 400 | 32 | 1 | 0 | 0 | 0 | 0 | 5 | 10 | 1 | 0 | 0 | 0 | Burkholderia cepacia | 1 |
| female | 1 | 210 | 94 | Anomalous Coronar | 1 | 0 | 5.9 | 6.45 | 15.9 | 144 | 51.1 | 30.7 | 640 | 680 | 0 | 1 | 0 | 2 | 1 | 7 | 28 | 1 | 0 | 0 | 0 | Burkholderia cepacia | 1 |
| female | 1 | 255 | 191 | Transposition of the Great Arteries | 1 | 0 | 4.1 | 17.7 | 6.4 | 74 | 32.9 | 32.7 | 1360 | 545 | 0 | 1 | 0 | 0 | 0 | 17 | 31 | 0 | 0 | 0 | 0 | 0 | 1 |
| male | 1 | 158 | 52 | Aortic Valve Sten | 1 | 1 | 3.2 | 15.1 | 4.9 | 80 | 51.9 | 28.5 | 395 | 210 | 0 | 1 | 0 | 0 | 0 | 6 | 22 | 1 | 0 | 0 | 0 | Klebsiella pneumoniae | 1 |
| male | 2 | 149 | 0 | Pulmonary Atresia | 1 | 0 | 9.2 | 16.3 | 9.5 | 38 | 9.8 | 44.2 | 595 | 318 | 1 | 0 | 0 | 1 | 0 | 5 | 18 | 1 | 0 | 0 | 0 | Klebsiella pneumoniae | 0 |
| male | 2 | 200 | 62 | Coarctation of the Aorta | 1 | 0 | 3.5 | 7.07 | 14.8 | 57 | 60.4 | 39 | 640 | 270 | 1 | 1 | 0 | 3 | 1 | 5 | 8 | 0 | 0 | 0 | 0 | 0 | 0 |
| female | 2 | 207 | 167 | Double Outlet Right Ventricle | 1 | 0 | 5.4 | 29.9 | 9.5 | 66 | 18.9 | 29.1 | 367 | 215 | 1 | 1 | 1 | 1 | 0 | 2 | 3 | 0 | 0 | 0 | 0 | 0 | 0 |
| female | 1 | 279 | 68 | Mitral Regurgitation | 1 | 0 | 11.1 | 3.37 | 32.6 | 238 | 339.3 | 31.2 | 500 | 700 | 0 | 1 | 1 | 0 | 0 | 31 | 32 | 0 | 0 | 0 | 0 | 0 | 0 |
| female | 1 | 253 | 52 | Single Ventricle | 1 | 0 | 12 | 28.07 | 5.8 | 40 | 8.5 | 40.8 | 600 | 280 | 0 | 0 | 0 | 0 | 0 | 5 | 17 | 0 | 0 | 0 | 0 | 0 | 1 |
| female | 2 | 212 | 136 | Transposition of the Great Arteries | 1 | 1 | 3.3 | 14.17 | 12.7 | 63 | 40.6 | 36.9 | 0 | 0 | 0 | 1 | 0 | 0 | 0 | 12 | 23 | 0 | 0 | 0 | 0 | 0 | 1 |
| male | 1 | 163 | 67 | Coarctation of the Aorta | 1 | 1 | 3.6 | 1.77 | 5 | 44 | 204.8 | 31.6 | 300 | 0 | 0 | 1 | 0 | 3 | 1 | 11 | 31 | 0 | 0 | 0 | 0 | 0 | 1 |
| female | 1 | 214 | 125 | Pulmonary Atresia | 1 | 0 | 15.9 | 13.37 | 6.2 | 77 | 74.3 | 32.5 | 1763 | 1393 | 0 | 1 | 0 | 1 | 1 | 7 | 35 | 0 | 0 | 0 | 0 | 0 | 0 |
| male | 1 | 259 | 99 | Interrupted Aortic Arch | 1 | 1 | 2.9 | 16.35 | 3.6 | 67 | 62.8 | 22.1 | 570 | 0 | 0 | 0 | 0 | 0 | 0 | 3 | 3 | 0 | 0 | 0 | 0 | 0 | 0 |
| female | 2 | 143 | 112 | tetralogy of Fallot | 1 | 0 | 5.6 | 4.3 | 4.2 | 26 | 6.7 | 45.9 | 400 | 0 | 0 | 1 | 0 | 1 | 0 | 7 | 20 | 0 | 0 | 0 | 0 | 0 | 1 |
| male | 2 | 306 | 156 | Pulmonary Atresia | 1 | 0 | 7 | 30.31 | 3.8 | 53 | 11.4 | 25.1 | 651 | 490 | 0 | 1 | 0 | 1 | 0 | 2 | 3 | 0 | 0 | 0 | 0 | 0 | 0 |
| female | 1 | 82 | 41 | Mitral Regurgitation | 1 | 0 | 26 | 6.17 | 10.6 | 52 | 24.4 | 34.2 | 450 | 700 | 0 | 0 | 0 | 0 | 0 | 9 | 9 | 0 | 0 | 0 | 0 | 0 | 0 |
| male | 2 | 118 | 83 | tetralogy of Fallot | 1 | 0 | 7.6 | 28.64 | 6.1 | 28 | 4.2 | 37.6 | 345 | 470 | 0 | 0 | 0 | 0 | 0 | 6 | 36 | 0 | 0 | 0 | 0 | 0 | 0 |
| female | 2 | 155 | 82 | Aortic Valve Sten | 1 | 0 | 16 | 1.57 | 9.9 | 69 | 16.6 | 21.5 | 155 | 170 | 0 | 1 | 0 | 0 | 0 | 10 | 10 | 0 | 0 | 0 | 0 | 0 | 0 |
| male | 1 | 222 | 155 | Transposition of the Great Arteries | 1 | 0 | 4.1 | 3.75 | 8 | 29 | 94.8 | 38.7 | 120 | 100 | 0 | 1 | 0 | 1 | 1 | 6 | 17 | 1 | 0 | 0 | 0 | Klebsiella pneumoniae | 1 |
| female | 1 | 185 | 76 | Coarctation of the Aorta | 1 | 0 | 5.6 | 2.4 | 3.6 | 21 | 16.2 | 32.3 | 205 | 50 | 0 | 1 | 0 | 5 | 1 | 7 | 57 | 0 | 0 | 0 | 0 | 0 | 1 |
| male | 1 | 280 | 87 | Atrioventricular | 1 | 0 | 4.8 | 3.49 | 2.6 | 34 | 39.3 | 34.9 | 100 | 140 | 0 | 1 | 0 | 0 | 0 | 3 | 3 | 0 | 0 | 0 | 0 | 0 | 0 |
| female | 2 | 276 | 129 | Ventricular Septa | 1 | 0 | 9.5 | 2.6 | 15 | 36 | 16.5 | 41.9 | 500 | 95 | 0 | 1 | 0 | 1 | 0 | 10 | 23 | 0 | 0 | 0 | 0 | 0 | 1 |
| male | 1 | 302 | 97 | Total Anomalous Pulmonary Venous | 1 | 0 | 3 | 17.4 | 8.5 | 29 | 17.4 | 34.7 | 232 | 190 | 0 | 1 | 0 | 1 | 1 | 12 | 18 | 1 | 0 | 0 | 0 | Stenotrophomonas maltophilia | 0 |
| male | 1 | 252 | 55 | Coarctation of the Aorta | 1 | 1 | 2 | 8.61 | 4.8 | 80 | 186.6 | 34.5 | 243 | 42 | 0 | 0 | 0 | 5 | 1 | 6 | 11 | 0 | 0 | 0 | 0 | 0 | 0 |
| female | 2 | 211 | 93 | Pulmonary Atresia | 1 | 0 | 7 | 11.46 | 4.3 | 22 | 3.9 | 46.1 | 170 | 50 | 0 | 1 | 1 | 2 | 0 | 27 | 29 | 0 | 0 | 0 | 0 | 0 | 0 |
| female | 2 | 323 | 187 | Transposition of the Great Arteries | 1 | 0 | 4.2 | 7.5 | 8.2 | 76 | 20.1 | 43.6 | 0 | 0 | 0 | 1 | 0 | 0 | 0 | 8 | 15 | 0 | 0 | 0 | 0 | 0 | 0 |
| male | 1 | 382 | 111 | Total Anomalous Pulmonary Venous | 1 | 1 | 2.9 | 14.43 | 1.5 | 32 | 111.8 | 24.5 | 250 | 80 | 0 | 1 | 0 | 0 | 0 | 6 | 8 | 0 | 0 | 0 | 0 | 0 | 0 |
| female | 1 | 183 | 105 | Interrupted Aortic Arch | 1 | 1 | 2.9 | 22.25 | 2.6 | 43 | 90.8 | 28.5 | 126 | 208 | 0 | 1 | 0 | 0 | 0 | 2 | 8 | 0 | 0 | 0 | 0 | 0 | 0 |
| male | 1 | 224 | 77 | Total Anomalous Pulmonary Venous | 1 | 1 | 3.5 | 3.8 | 9.4 | 58 | 62.5 | 34 | 100 | 105 | 0 | 1 | 0 | 2 | 1 | 8 | 68 | 1 | 0 | 0 | 0 | Streptococcus mitis | 1 |
| male | 1 | 202 | 86 | Total Anomalous Pulmonary Venous | 1 | 1 | 3 | 12.88 | 1.2 | 43 | 184.8 | 45.8 | 190 | 60 | 0 | 1 | 0 | 1 | 0 | 2 | 6 | 0 | 0 | 0 | 0 | 0 | 0 |
| female | 2 | 110 | 97 | Total Anomalous Pulmonary Venous | 1 | 0 | 6.9 | 7.33 | 4.7 | 33 | 29.3 | 46.7 | 60 | 30 | 0 | 0 | 0 | 1 | 0 | 8 | 21 | 1 | 0 | 0 | 0 | Pseudomonas aeruginosa | 1 |
| male | 2 | 302 | 204 | Mitral Regurgitation | 1 | 0 | 9 | 9.32 | 12.5 | 46 | 60.1 | 41.2 | 350 | 40 | 0 | 0 | 0 | 0 | 0 | 4 | 4 | 0 | 0 | 0 | 0 | 0 | 0 |
| male | 2 | 230 | 157 | Transposition of the Great Arteries | 1 | 1 | 3.4 | 29.15 | 9.8 | 81 | 80.1 | 17.6 | 225 | 15 | 0 | 1 | 1 | 5 | 1 | 7 | 19 | 0 | 0 | 0 | 0 | 0 | 0 |
| female | 1 | 349 | 161 | Transposition of the Great Arteries | 1 | 0 | 2.8 | 11.31 | 15.9 | 59 | 46 | 35.2 | 130 | 100 | 0 | 1 | 0 | 3 | 1 | 2 | 11 | 1 | 0 | 0 | 0 | Streptococcus pneumoniae | 0 |
| male | 2 | 151 | 88 | Total Anomalous Pulmonary Venous | 1 | 0 | 4.5 | 13 | 8.9 | 92 | 47 | 33.9 | 406 | 130 | 0 | 1 | 0 | 3 | 0 | 7 | 22 | 0 | 0 | 0 | 0 | 0 | 1 |
| female | 2 | 361 | 104 | Total Anomalous Pulmonary Venous | 1 | 0 | 4.8 | 11.41 | 6.6 | 50 | 15.6 | 31.6 | 210 | 110 | 0 | 0 | 0 | 5 | 1 | 13 | 46 | 1 | 1 | 0 | 0 | Staphylococcus epidermidis | 0 |
| male | 1 | 526 | 148 | Double Outlet Right Ventricle | 1 | 0 | 3.8 | 8 | 11.4 | 75 | 72.4 | 27.3 | 530 | 150 | 0 | 0 | 1 | 0 | 0 | 8 | 27 | 0 | 0 | 0 | 0 | 0 | 0 |
| male | 1 | 197 | 184 | Pulmonary Atresia | 1 | 0 | 4.5 | 4.27 | 8.7 | 38 | 35.2 | 34 | 70 | 5 | 0 | 1 | 0 | 0 | 0 | 8 | 25 | 0 | 0 | 0 | 0 | 0 | 1 |
| male | 1 | 337 | 155 | Transposition of the Great Arteries | 1 | 1 | 3.5 | 10.3 | 4.6 | 121 | 33.9 | 19.2 | 0 | 0 | 0 | 1 | 0 | 1 | 1 | 2 | 3 | 0 | 0 | 0 | 0 | 0 | 0 |
| female | 2 | 265 | 65 | Mitral Regurgitation | 1 | 0 | 27.5 | 5.34 | 12.9 | 66 | 64.1 | 41.9 | 200 | 100 | 0 | 0 | 0 | 0 | 0 | 11 | 17 | 0 | 0 | 0 | 0 | 0 | 1 |
| male | 1 | 199 | 55 | Coarctation of the Aorta | 1 | 1 | 3.1 | 6.3 | 8.4 | 92 | 94.4 | 15 | 0 | 0 | 0 | 1 | 0 | 0 | 0 | 2 | 2 | 0 | 0 | 0 | 0 | 0 | 0 |
| male | 1 | 346 | 146 | Total Anomalous Pulmonary Venous | 1 | 1 | 3.5 | 7.88 | 4.1 | 40 | 221 | 35.3 | 140 | 50 | 0 | 1 | 0 | 0 | 0 | 7 | 14 | 0 | 0 | 0 | 0 | 0 | 1 |
| male | 1 | 600 | 360 | Anomalous Coronar | 1 | 0 | 6.3 | 5.57 | 10.6 | 43 | 35 | 40.7 | 300 | 108 | 0 | 0 | 0 | 0 | 0 | 11 | 11 | 0 | 0 | 0 | 0 | 0 | 1 |
| female | 1 | 420 | 71 | Pulmonary Atresia | 1 | 0 | 9 | 4.3 | 8.9 | 50 | 36.7 | 28.2 | 150 | 85 | 0 | 1 | 0 | 0 | 0 | 13 | 275 | 1 | 1 | 0 | 0 | Stenotrophomonas maltophilia , Staphylococcus epidermidis | 1 |
| female | 1 | 284 | 88 | Double Outlet Right Ventricle | 0 | 0 | 44 | 6.84 | 9.1 | 104 | 63.6 | 21.8 | 1443 | 373 | 0 | 1 | 1 | 0 | 0 | 4 | 64 | 0 | 0 | 0 | 0 | 0 | 1 |
| male | 1 | 286 | 78 | Total Anomalous Pulmonary Venous | 1 | 0 | 2.7 | 10.7 | 4.3 | 81 | 69.1 | 33.4 | 240 | 90 | 0 | 1 | 0 | 0 | 0 | 11 | 18 | 0 | 0 | 0 | 0 | 0 | 1 |
| female | 2 | 102 | 80 | tetralogy of Fallot | 1 | 0 | 2.4 | 14.3 | 12.8 | 59 | 17 | 39 | 160 | 40 | 0 | 1 | 0 | 7 | 1 | 13 | 20 | 0 | 0 | 0 | 0 | 0 | 0 |
| male | 2 | 125 | 93 | tetralogy of Fallot | 1 | 0 | 6.2 | 1.54 | 14.1 | 26 | 21.6 | 48.9 | 205 | 0 | 0 | 1 | 0 | 10 | 1 | 29 | 54 | 1 | 1 | 0 | 0 | Staphylococcus aureus | 0 |
| female | 2 | 0 | 0 | Thoracotomy for E | 0 | 0 | 18 | 17.87 | 16.4 | 88 | 22.6 | 28.6 | 160 | 0 | 0 | 0 | 1 | 0 | 0 | 14 | 14 | 1 | 0 | 0 | 0 | Acinetobacter baumannii, Pseudomonas aeruginosa | 0 |
| male | 2 | 116 | 95 | tetralogy of Fallot | 0 | 0 | 8.9 | 7.13 | 22.2 | 172 | 19.1 | 36.8 | 250 | 15 | 0 | 1 | 0 | 11 | 1 | 20 | 36 | 1 | 0 | 0 | 0 | Stenotrophomonas maltophilia | 0 |
| male | 2 | 110 | 53 | Interrupted Aortic Arch | 1 | 1 | 2.8 | 24.63 | 7 | 161 | 105.3 | 34.4 | 258 | 60 | 1 | 1 | 0 | 1 | 0 | 7 | 8 | 0 | 0 | 0 | 0 | 0 | 0 |
