# Supplementary material for: Risk factors for healthcare-associated infections in children undergoing ECMO after cardiac surgery for congenital heart disease: A retrospective study
Source: PLoS One. 2026 May 11;21(5):e0348220. doi: 10.1371/journal.pone.0348220 (PMC13160298; doi:10.1371/journal.pone.0348220)
Supplement: Attachment — Submitted filename: Response to Reviewers.docx [file pone.0348220.s003.docx]

**Rviewers1 comment:** My main concern is the definition of infection in the patient population - while positive respiratory cultures generally translates into infection, it could also be colonization. It would be interesting to look at the chest imaging and ECMO settings along with laboratory results to determine if they were real infections or not. On the other hand, the authors should also acknowledge the fact that positive cultures alone may underestimate clinical infections if antibiotics were used prior to cannulation.
I also recommend to discuss potential confounding when it comes to ICU length of stay - a time-to-event analysis could be implemented.

**Response:** Thank you very much for your constructive feedback.

1、During the ECMO support process in children, the diagnosis of infection is relatively difficult. Due to the presence of the cooling water tank and the systemic inflammatory response caused by the contact between blood and foreign substances (extracorporeal circulation and ECMO-related pipelines), it cannot be clearly diagnosed through clinical signs (fever), laboratory tests (white blood cells, C-reactive protein, procalcitonin) and chest X-ray findings. Pulmonary injuries after cardiac surgery, such as pulmonary edema and atelectasis, can also affect the diagnosis of infection.In this study, only microbial indicators were used as the indicators of infection, and this diagnostic standard has been widely adopted and applied.

2、For pediatric patients with relatively high infection indicators, antibiotics may be administered as appropriate before cannulation. This can lead to negative culture results, resulting in an underestimation of clinical infections.

3、It has been supplemented.

The length of ICU stay includes pre - operative ICU stay and post - operative ICU stay. If a child is in a stable condition before surgery and does not require endotracheal intubation, they will not be transferred to the ICU, resulting in a relatively shorter overall ICU stay. Conversely, if a child needs endotracheal intubation before surgery, the total ICU stay will be prolonged. On the other hand, children who require pre - operative endotracheal intubation are usually in more severe conditions, leading to a longer post - operative recovery period and thus an extended post - operative ICU stay. In other words, pre - operative endotracheal intubation will increase the length of children's ICU stay.

**Reviewer2**

The retrospective single-center design with a relatively small cohort (n=54) limits the generalizability of the findings. Please expand the discussion on how the limited sample size may affect the robustness of the logistic regression analysis. The authors rely exclusively on microbiological confirmation, without considering clinical and biochemical markers (e.g., CRP, PCT). This approach could underestimate clinically relevant infections. A comparison with alternative diagnostic criteria would strengthen the manuscript. In univariate analysis, both ICU stay and pre-ECMO ventilation duration showed associations with infection, but they lost significance in multivariate analysis. Please comment on possible interactions between these variables and preoperative intubation. The conclusion emphasizes avoiding preoperative intubation when possible. The authors should provide more practical recommendations (e.g., airway management strategies, antimicrobial prophylaxis protocols, VAP prevention bundles) to enhance clinical applicability.

**Response:** Thanks for your key comment! We have carefully addressed your valuable suggestions as follows：

1、The main risks of insufficient sample size (failing to meet the Events Per Variable≥10, EPV) for Logistic regression are the phenomena of complete separation and quasi-complete separation. In terms of results, these risks manifest as either the inability to obtain the regression coefficient B, or an excessively large Wald (resulting in an extremely small p-value). However, in this analysis, the regression coefficient *B* is 1.601, with a Wald of 4.43 (p=0.035), and neither complete separation nor quasi-complete separation is observed.

It was a retrospective single center study using observationsal analysis. The sample size of this study was small, which does not meet the requirements of events per variable. However, considering the relatively small number of ECMO-Associated Nosocomial Infections in children after congenital heart disease surgery, the results are valuable. However, the reliability of this result needs further confirmation.

2、During the ECMO support process in children, the diagnosis of infection is relatively difficult. Due to the presence of the cooling water tank and the systemic inflammatory response caused by the contact between blood and foreign substances (extracorporeal circulation and ECMO-related pipelines), it cannot be clearly diagnosed through clinical signs (fever), laboratory tests (white blood cells, C-reactive protein, procalcitonin) and chest X-ray findings. In this study, only microbial indicators were used as the indicators of infection, and this diagnostic standard has been widely adopted and applied.For pediatric patients with relatively high infection indicators, antibiotics may be administered as appropriate before cannulation. This can lead to negative culture results, resulting in an underestimation of clinical infections.

3、Pediatric patients requiring preoperative endotracheal intubation are in critical condition, which will increase their post-operative ICU length of stay. The subjects of our study are pediatric patients receiving VA ECMO support after cardiac surgery; preoperative intubation will inevitably lead to prolonged pre-ECMO ventilation duration.

4、The specific recommendations are as follows:

(1) Strict preoperative evaluation of intubation indications, with efforts to minimize the time interval between intubation and surgery.
(2) Meticulous aseptic technique during intubation and intensified postoperative airway management.Use an airway filter to assist with humidification while preventing airway contamination. Strictly adhere to aseptic techniques: wash hands and wear sterile gloves before suctioning, use disposable suction catheters to avoid cross-infection; administer 100% oxygen for 30 seconds prior to suctioning to prevent transient hypoxemia induced by suctioning from exacerbating cardiac burden.
(3) Close monitoring of infection markers during ECMO, with prophylactic antimicrobial strategies for high-risk patients.Antibiotics should only be used for pediatric patients with clear high-risk factors for infection before surgery, such as pre-existing pulmonary infection (chest X-ray indicating inflammation, elevated white blood cell count/CRP in blood routine).For patients without clear high-risk factors, routine prophylactic use of antibiotics before surgery is not recommended; only strict aseptic techniques should be implemented during intubation to reduce the risk of iatrogenic infection.Broad-spectrum antibiotics are preferred, and specific selections should be adjusted based on the hospital's bacterial resistance surveillance data.
(4) Ventilator-Associated Pneumonia (VAP) Prevention Measures: During non-surgical periods, elevate the head of the bed by 30°-45° to reduce reflux and aspiration of gastric contents, thereby lowering the risk of VAP. Perform oral care every 6 hours to inhibit the colonization of pathogenic bacteria in the oral cavity and reduce retrograde airway infection caused by bacteria. Ventilator circuits: Replace every 7 days; if contamination (e.g., sputum contamination) occurs, replace immediately. Drain condensed water in a timely manner to prevent it from refluxing into the airway or humidifier.
