# Supplementary material for: Risk factors for healthcare-associated infections in children undergoing ECMO after cardiac surgery for congenital heart disease: A retrospective study
Source: PLoS One. 2026 May 11;21(5):e0348220. doi: 10.1371/journal.pone.0348220 (PMC13160298; doi:10.1371/journal.pone.0348220)
Supplement: Attachment — Submitted filename: Response_to_Reviewers_auresp_2.docx [file pone.0348220.s004.docx]

**Response to Reviewer #2:** Thank you very much for your constructive feedback. We have carefully addressed your valuable suggestions as follows:

1. **Statistical limitations The authors added a paragraph on the limitations of small sample size for logistic regression. It would be useful to explicitly report the events-per-variable ratio (EPV) calculated for this model to quantify the degree of statistical limitation.**

**Response :**In this study, there were 14 infected patients. Multivariate logistic regression analysis included 2 predictive variables(EPV=7) . The results of standard logistic regression analysis are usually reliable and have certain clinical guiding significance.

2. **Diagnostic criteria paragraph The justification for microbiological-only diagnosis is sound, but please clarify whether negative cultures with high clinical suspicion (e.g., prior antibiotics) were recorded or excluded. This will help readers interpret potential underestimation bias.**

**Response :**For children with clinically highly suspected infection but negative culture results, we empirically administer antibiotics; however, such children were not included in the infection group in this study.

3. **Terminology consistency Use consistent terminology throughout: “ECMO-associated nosocomial infection” vs. “ECMO-related infection.” Consider harmonizing the terms in title, abstract, and text.**

**Response :**Modified.

4. **Discussion on causality The manuscript mentions the possible bidirectional relationship between ICU stay and infection. It would strengthen the discussion to acknowledge that reverse causality cannot be excluded in this retrospective design.**

**Response :**  The length of ICU stay includes pre - operative ICU stay and post - operative ICU stay. If a child is in a stable condition before surgery and does not require endotracheal intubation, they will not be transferred to the ICU, resulting in a relatively shorter ICU stay. Conversely, if a child needs endotracheal intubation before surgery, the total ICU stay will be prolonged. Relevant studies have indicated that infections following congenital heart disease surgery prolong the length of ICU stay, while preoperative ICU admission is one of the risk factors for developing infections. This is largely consistent with our research—all children who underwent preoperative endotracheal intubation were admitted to the ICU.On the other hand, the preoperative ICU stay duration also increases the total ICU length of stay for children and elevates their risk of infection.A shorter ICU stay in the non-infected group could be related to treatment withdrawal due to disease severity or financial constraints, suggesting a potential bidirectional relationship. Further studies are needed to clarify this causal association.

**Response to Reviewer #3:** Thank you very much for your constructive feedback. We have carefully addressed your valuable suggestions as follows:

1. **Title The expression “nosocomial infection” is outdated. I recommend replacing it with the term “healthcare-associated infection (HAI)”, as endorsed by the World Health Organization (WHO) and CDC. Consider including the study design in the title, following STROBE recommendations for observational studies.**

**Response :**Modified.

2. **Abstract The objective stated in the abstract does not match the one described in the introduction; these must be harmonized to ensure consistency throughout the paper. Please clarify the study design explicitly (case-control or cohort). The current abstract does not mention it. Include a clear statement of main findings and conclusion that aligns with the results.**

**Response :**Modified. Retrospective cohort study.

3. **Introduction The introduction is overly brief. It should better contextualize the relevance of infections during ECMO, with updated epidemiological data and a clearer definition of the literature gap your study addresses. The objective should directly reflect the research question and align with both the abstract and methods.**

**Response :**Modified.

4. **Methods The methods section requires substantial expansion to meet STROBE and EQUATOR Network standards for transparency. Please specify: The setting of postoperative care (confirm if all patients were managed in an ICU). Whether the center is ELSO-certified, and how ECMO management is organized (nurse-led, perfusionist-led, or mixed). The nurse-to-patient ratio or staffing model, as these factors influence infection risk. The study design is described as “case-control”, but the approach appears more consistent with a retrospective cohort, since patients were divided according to infection occurrence. Please clarify. The definition of healthcare-associated infection appears to rely on outdated criteria (2009). Use current WHO or CDC definitions, and describe how each infection type (bloodstream, respiratory, urinary, etc.) was diagnosed. Indicate: Who was responsible for data collection and on which platform data were stored. How patient confidentiality and anonymization were ensured. Any bias-control procedures applied during data review. In the statistical analysis, describe: Criteria for variable selection in the regression model. Quality metrics (R², AUC, VIF, Hosmer-Lemeshow, etc.). How missing data were handled.**

**Response :**Modified.

All children were treated in the ICU and managed jointly by ECMO-qualified doctors, nurses, and perfusionists. Based on the children's chest X-ray findings and laboratory test results, empirical antibiotic therapy was administered when infection was highly suspected, and the antibiotic regimen was adjusted after the results of bacterial culture and drug sensitivity test were obtained.Multivariate logistic regression analysis was employed to identify independent risk factors for ECMO-related healthcare-associated infections following congenital heart surgery.If the EPV (Events Per Variable) is less than 5, Firth logistic regression analysis should be adopted to reduce the risk of overfitting in small samples. All data has been uploaded to the journal's website!

5. **Results Please include a flow diagram or statement indicating the number of patients screened, included, and excluded, as recommended by STROBE. Tables should be reformatted for consistency — avoid box-style formatting, ensure uniform fonts, and standardize units of measurement across variables. Some numerical results lack dispersion measures (SD or IQR); please include them.**

**Response :**Modified.

A flow diagram has been established.Normally distributed continuous variables were expressed as mean ± standard deviation and compared using Student's t-test. Non-normally distributed continuous variables were presented as median (interquartile range) [M(Q1,Q3)] and analyzed using the Mann-Whitney U test. **Categorical variables were expressed as frequencies (percentages) and compared using either chi-square test or Fisher's exact test, as appropriate.**

6. **Discussion The discussion should more thoroughly address your main finding — the association between preoperative intubation and ventilator-associated infection. Expand the discussion by comparing your results to recent studies and highlighting how your findings add to or differ from existing evidence. The implications section currently appears as bullet points and lacks supporting references; please reframe it into a narrative form with appropriate citations.**

**Response：** Modified.

A meta-analysis involving over 50,000 pediatric patients found that infection had the highest incidence among tracheal intubation-related complications, reaching 11.9%[17].Relevant studies have indicated that infections following congenital heart disease surgery prolong the length of ICU stay, while preoperative ICU admission is one of the risk factors for developing infections.[25]. This is largely consistent with our research—all children who underwent preoperative endotracheal intubation were admitted to the ICU.On the other hand, the preoperative ICU stay duration also increases the total ICU length of stay for children and elevates their risk of infection.

7. **Conclusion Present the conclusion as a separate section, summarizing the main findings, their implications for practice, and limitations.**

**Response :**Modified.

Limitations of this study include the inherent biases of retrospective design, potential constraints on generalizability due to single-center data, and lack of stratified analysis of pathogen types and antibiotic usage patterns. Future multicenter prospective studies incorporating microbiological evidence and immune function monitoring are needed to further elucidate the pathological mechanisms of ECMO-associated infections and optimize prevention strategies.In this study, there were 14 infected patients. Multivariate logistic regression analysis included 2 predictive variables(EPV=7) . The results of standard logistic regression analysis are usually reliable and have certain clinical guiding significance.

In summary, healthcare-associated infections during ECMO support following pediatric congenital heart surgery were predominantly Gram-negative respiratory infections. Preoperative endotracheal intubation was identified as an independent risk factor for these infections, suggesting that minimizing pre-operative intubation in congenital heart disease patients may reduce infection rates. These findings highlight the need for clinicians to carefully weigh the necessity of intubation against infection risks when managing critically ill children with congenital heart disease, while implementing enhanced infection control measures.

**8. References Only about 30% of the references are from the last five years. Updating the literature will strengthen the scientific relevance of the manuscript. I suggest including recent ECMO infection studies (2020–2024), particularly from ELSO centers.**

**Response :**Modified.Six of the latest references have been added or updated.
