# Supplementary material for: Risk factors for healthcare-associated infections in children undergoing ECMO after cardiac surgery for congenital heart disease: A retrospective study
Source: PLoS One. 2026 May 11;21(5):e0348220. doi: 10.1371/journal.pone.0348220 (PMC13160298; doi:10.1371/journal.pone.0348220)
Supplement: Attachment — Submitted filename: Response_to_Reviewers_auresp_3.docx [file pone.0348220.s005.docx]

**Response to Reviewer #2:** Thank you very much for your constructive feedback. We have carefully addressed your valuable suggestions as follows:

1) Thank you for providing the specific EPV ratio of 7 in your response, which technically addresses the initial query regarding statistical limitations. As this value is above the critical threshold of 5, the use of standard logistic regression is methodologically defensible. However, for maximum scientific rigor and interpretative transparency, I must highlight that the 95% CI for the primary independent risk factor (preoperative endotracheal intubation: OR=4.852, 95% CI: 1.174–20.059) remains exceptionally wide. This broad CI is a clear statistical manifestation of the imprecision and limited statistical power inherent to the small sample size (n=54) and the low number of events (n=14). While the analysis is technically valid, the wide range indicates significant uncertainty, confirming that the estimate is not precise. I strongly recommend the authors integrate an explicit caution into the Discussion/Limitations section, acknowledging that despite the EPV being technically acceptable, the resultant broad confidence interval necessitates a careful and cautious interpretation of the multivariate findings. This is not a critique of the methodology, but a crucial measure to enhance the scientific rigor of the interpretation.
   Additionally, the statement in the Methods section regarding the contingency plan to use Firth logistic regression " If the EPV (Events Per Variable) is less than 5, Firth logistic regression analysis should be adopted to reduce the risk of overfitting in small samples” describes a procedure that was ultimately not executed, as the reported EPV was 7. Since the Methods section should strictly detail the procedures that were actually performed, this sentence should be removed from the Statistical Analysis subsection. The calculation of an EPV of 7 and the subsequent use of standard logistic regression should instead be referenced in the Results, noting that the sample size constraints are already appropriately discussed in the Limitations section.

**Response :**Discussion section has been modified. 1、 As EPV is above the critical threshold of 5, the use of standard logistic regression is methodologically defensible. However, for maximum scientific rigor and interpretative transparency, I must highlight that the 95% CI for the primary independent risk factor (preoperative endotracheal intubation: OR=4.852, 95% CI: 1.174–20.059) remains exceptionally wide. 2、the statement in the Methods section has been modified.

2) Thank you for clarifying the strict case definition used for the infection group. The decision to exclude children with clinically highly suspected infection but negative culture results (despite receiving empirical antibiotics) from the primary infection group provides methodological consistency by relying solely on microbiological confirmation. This approach is transparent and helps mitigate the risk of misdiagnosis in the complex context of ECMO-related systemic inflammation.
However, for maximum transparency and to aid readers in interpreting potential biases, two actions are required for the final manuscript:
1) This critical exclusion criterion must be explicitly stated within the Methods section (specifically, under the data collection or diagnostic criteria subheading). It cannot remain solely in the response to reviewers.
2) Given that children with high clinical suspicion who received antibiotics were excluded, there is a potential for underestimation bias regarding the true incidence of infection (especially due to the suppression of bacterial growth from prior antimicrobial therapy). I strongly recommend the authors explicitly acknowledge this potential underestimation bias in the Discussion/Limitations section of the manuscript.

**Response** :1、Added.All children were treated in the ICU and managed jointly by ECMO-qualified doctors, nurses, and perfusionists. Based on the children's chest X-ray findings and laboratory test results, empirical antibiotic therapy was administered when infection was highly suspected, and the antibiotic regimen was adjusted after the results of bacterial culture and drug sensitivity test were obtained.

2、Discussion section has been modified.Due to the diagnostic criteria, children with a high suspicion of infection, but negative culture, were classified as non-infected, which would underestimate the incidence HAI.

3) While the authors responded that the inconsistent terminology was "Modified", a meticulous review reveals that the implementation of the agreed-upon term "healthcare-associated infection (HAI)" has been incomplete. This incomplete harmonization constitutes an essential editorial failure that must be corrected before acceptance. The term "nosocomial infection" or variations thereof must be entirely replaced by "healthcare-associated infection (HAI)" throughout the manuscript.
Specifically, the following instances of the outdated term persist:
1. Inconsistencies within the main text:
- Methods/Exclusion Criteria: The exclusion criterion still refers to the "Presence of nosocomial infection before ECMO initiation". This must be changed to "healthcare-associated infection".
- Results/Multivariate Analysis: The sentence describing the key finding uses the phrase: "Variables with p < 0.05 in the univariate analysis were included in the multivariate logistic regression analysis, which revealed that preoperative endotracheal intubation in children with congenital heart disease (OR = 4.852, 95% CI: 1.174-20.059; p = 0.029) was an independent risk factor for nosocomial ECMO-related infections in pediatric postcardiotomy patients." This must be harmonized to "ECMO-related healthcare-associated infections”.
2. Inconsistencies within Tables and Figures
- Table 1 Title: The title of Table 1 remains "Pathogenic Microorganisms of ECMO-Associated Nosocomial Infections in children...".
- Table 2 Title: The title of Table 2 remains "Univariate Analysis of ECMO-Associated Nosocomial Infections in children...".
The authors must perform a final, comprehensive search-and-replace to ensure that every instance of "nosocomial infection" (including text, definitions, tables, and figure captions) is harmonized with "healthcare-associated infection (HAI)" to ensure full editorial and scientific consistency as agreed.

**Response :**Modified.

**Response to Reviewer #3:** Thank you very much for your constructive feedback. We have carefully addressed your valuable suggestions as follows:

1) As previously noted (in the detailed response to Reviewer 2, Point 3), the outdated term "nosocomial infection" persists in crucial parts of the manuscript, despite the authors' claim that the terminology was "Modified."

This persistent error is evident in the titles of Table 1 and Table 2, the exclusion criteria, and the Results section. For the sake of scientific consistency, the authors must perform a final correction to replace this term with the modern "healthcare-associated infection (HAI)" everywhere.

Furthermore, the authors successfully implemented the structural requirement of including the study design in the title, following STROBE recommendations for observational studies, which is now acceptable.

**Response :**Modified.

2) The authors have successfully revised the Abstract to ensure its objective is harmonized with the Introduction. Furthermore, the study design is now explicitly stated as a "retrospective cohort study", and the Abstract includes a clear summary of the main statistical findings and conclusions.

**Response :**Thanks.

3) The authors successfully expanded the Introduction to provide better context and relevance. They incorporated updated epidemiological data and clearly defined the literature gap regarding risk factors for HAI in pediatric post-cardiac surgery ECMO patients. Furthermore, the objective of the study is now clearly stated and aligned with the Abstract and Methods sections, making the Introduction logically sound and comprehensive.

**Response :**Thanks.

4) as previously requested by the reviewer 3, the authors should explicitly state whether the Fuwai Central China Cardiovascular Hospital ECMO Center holds official ELSO certification.

The authors must provide the typical nurse-to-patient ratio in the Pediatric ICU where these patients were managed, as this directly affects the generalizability of infection control findings. Additionally, please provide a brief statement detailing who collected the data, the original storage method, and the confidentiality/anonymization procedures used.

The authors should report at least one quality metric for the final multivariate logistic regression model in the Results section to demonstrate model fit and robustness. Additionally, please specify the approach used for missing data, or declare if no variables had missing data.

**Response :**Our hospital is a nationally accredited ECMO Quality Control Center, with a nurse-to-patient ratio of 1.9 in the pediatric intensive care unit.No variables included in the final analysis had missing data.

5) The authors have substantially resolved the methodological requirements for the Results section: a flow diagram (Figure 1) has been established, and the authors clarified the correct use of mean ± SD versus Median (IQR) for data presentation in the Material and Methods section. However, the editorial implementation remains incomplete, as previously noted: the obsolete term "Nosocomial Infections" persists in the titles of Table 1 and Table 2, constituting a critical inconsistency that must be corrected. Furthermore, for maximal clarity, the authors must ensure that units of measurement, utilized fonts, and the use of capitalization and lowercase letters are uniform throughout the tables, and that a clear description of the format used for variable descriptions is formally stated in the table caption to fully satisfy the requirements for data presentation rigor. In addition, I recommend consistently using the lowercase p of p-value (i.e., “p-value" instead of "P-value") throughout the manuscript, as this is the preferred convention in formal statistical reporting.

**Response :**Modified.

6) The authors have successfully expanded the Discussion section, achieving the required standard. They provided deeper mechanistic insight into the association between preoperative intubation and HAI risk, included comparisons with recent relevant meta-analyses, and fully converted the implications section into a narrative format.

**Response :**Thanks.

7) The authors have successfully presented the Conclusion as a dedicated, separate section that fully complies with the request. It provides a concise summary of the main findings, clinical implications, necessary future studies, and explicitly details the study's limitations.

**Response :**Thanks.

8) The authors have adequately updated the bibliography with six highly relevant, recent references (2023–2025), including ELSO registry data, substantially strengthening the manuscript's scientific relevance.

**Response :**Thanks.

A comprehensive review of the tables and figures reveals several unresolved issues regarding formatting, statistical reporting consistency, and terminology that must be addressed before final acceptance.

**1.** Global formatting and consistency issues (Tables 1, 2, and 3)

- The obsolete term "Nosocomial Infections" persists in the titles of Table 1 and Table 2. This must be corrected to "Healthcare-associated Infections (HAI)" for full manuscript consistency.

- The use of capitalization and lowercase letters must be uniform across all tables.

- The separators used in text (e.g., "/" in "Gender (male/female)" versus "vs." in other parts of the text) should be standardized across the entire manuscript for uniform style.

- The authors must ensure that a clear description of the data format (e.g., Mean ± SD or Median (IQR)) is formally stated in the table caption for clarity.

**Response** :Modified.A clear description of the data format (**[**$\bar{\text{x}}$±s,M(Q1,Q3),n(%)]) is formally stated in the table caption for clarity.

2. Table 2: Univariate analysis specific issues

- For the univariate analysis of the Admission diagnosis variable (row “admission diagnosis/n”), only a pooled p-value (0.318) is reported, and the corresponding X2 statistic is not shown. Given the very small cell counts for several diagnostic categories in the infected group, the authors must clarify in the Methods section whether these categories were grouped or collapsed before applying the Chi-square test. They also should systematically review all categorical variables in Table 2. Wherever expected cell frequencies are <5 in more than 20% of cells, the p-values must be recalculated using Fisher’s Exact Test, and any updated values should be clearly reported.

- While the text mentions variables were measured "before extracorporeal membrane oxygenation (ECMO)", the column headers for biochemical values (lactate, BUN, creatinine, bilirubin, albumin) should be explicitly labeled (e.g., "Pre-ECMO Blood lactate") to eliminate any ambiguity.

**Response** :Modified.Given the very small cell counts for several diagnostic categories in the infected group, these categories were grouped before applying the Chi-square test.These were divided into two groups.Wherever expected cell frequencies are <5 in more than 20% of cells, the *p*-values must be recalculated using Fisher’s Exact Test.

3. Figure 1: Flow Diagram

- The current figure caption "Fig1: Healthcare-associated Infections of ECMO in Children After congenital heart disease Surgery" is overly descriptive. Please revise it to a standard formal title such as "Flow diagram of patient selection"

**Response :**Modified.Flow diagram of patient selection
