# Supplementary material for: Risk factors for healthcare-associated infections in children undergoing ECMO after cardiac surgery for congenital heart disease: A retrospective study
Source: PLoS One. 2026 May 11;21(5):e0348220. doi: 10.1371/journal.pone.0348220 (PMC13160298; doi:10.1371/journal.pone.0348220)
Supplement: Attachment — Submitted filename: Response_to_Reviewers_auresp_4.docx [file pone.0348220.s006.docx]

**Response to Reviewer #4:** Thank you very much for your constructive feedback. We have carefully addressed your valuable suggestions as follows:

1- The statement regarding the contingency plan to use Firth logistic regression ("If the EPV... is less than 5, Firth logistic regression analysis should be adopted...") still remains in the Statistical Analysis subsection of the Methods. As previously noted, the Methods section must strictly reflect the procedures actually performed. Since your reported EPV was 7, this sentence must be removed.

**Response :**Modified.Deleted.

2- The discussion regarding the wide 95% Confidence Interval (95% CI: 1.174–20.059) for preoperative endotracheal intubation has been inserted into the Discussion/Limitations section almost verbatim as a response to a reviewer, using phrases like "I must highlight that...". This should be rewritten as an integrated methodological limitation in the authors' own voice, explicitly acknowledging that the broad CI is a manifestation of limited statistical power and small sample size.

**Response :**Modified.Although the EPV ratio (>5) justifies the use of standard logistic regression, it is important to acknowledge that the 95% Confidence Interval for preoperative endotracheal intubation (OR=4.852, 95% CI: 1.174–20.059) remains wide. This width suggests a degree of estimation uncertainty, likely attributable to the limited number of events within this specific subgroup, and warrants a cautious interpretation of the magnitude of the risk.

3- In the Discussion, the claim that the results are "usually reliable" tends to downplay the significant uncertainty indicated by the wide confidence interval previously highlighted. Please ensure the tone reflects the necessary scientific caution requested.

**Response :**Modified.

4- You have added the explanation regarding empirical antibiotic therapy and the exclusion of culture-negative patients in two different sections (Data Collection and ECMO Management). Please consolidate this information into a single, clear paragraph. It is vital to explicitly state—without ambiguity—that microbiological confirmation was a mandatory requirement for the "infection group" to maintain the study's specificity.

**Response :**Modified. Based on the children's chest X-ray findings and laboratory test results, empirical antibiotic therapy was administered when infection was highly suspected, and the antibiotic regimen was adjusted after the results of bacterial culture and drug sensitivity test were obtained.

5- The mention of underestimation bias in the Limitations section remains somewhat superficial. I recommend expanding this to explicitly state that prior antimicrobial therapy might have suppressed bacterial growth, leading to false-negative cultures, which is a common challenge in ECMO-related HAI diagnosis.

**Response :**Modified. I explicitly state that prior antimicrobial therapy might have suppressed bacterial growth, leading to false-negative cultures, which is a common challenge in ECMO-related HAI diagnosis.

**Response to Reviewer #5:** Thank you very much for your constructive feedback. We have carefully addressed your valuable suggestions as follows:

1 Ethical Transparency & Methodology.

a. Data Integrity Statement (Ignored): The request to detail who collected the data, storage methods, and anonymization procedures was ignored. You must add this to the Methods section to comply with ethical standards.

**Response :**Modified. We thank the reviewer for highlighting this critical oversight regarding data integrity and ethical compliance. We agree that detailing data collection, storage, and anonymization procedures is essential for scientific transparency. In response, we have now added a "Data Collection and Management" subsection to the Methods section. This new passage explicitly describes that data were collected by two trained researchers from electronic medical records, stored in a password-protected database, and fully anonymized by removing personal identifiers and assigning unique study codes prior to analysis. We believe this addition addresses the reviewer's concern and aligns our manuscript with standard ethical reporting guidelines.

b. Evaluation of Model Performance and Fit: The authors present the results of the multivariate logistic regression solely in terms of Odds Ratios and p-values. However, providing coefficient estimates without assessing the overall performance of the model is methodologically insufficient, particularly given the small sample size (n=54) and the low number of events (n=14).In small datasets, logistic regression models are prone to overfitting, where the model describes random error rather than underlying relationships. To demonstrate the robustness of the findings, the authors must report:

Discrimination: The C-statistic (or Area Under the ROC Curve - AUC), which indicates the model's ability to distinguish between patients who developed an infection and those who did not. An OR of 4.85 is of limited value if the model's discriminative ability is poor (e.g., AUC < 0.7).

Calibration (Goodness-of-Fit): A measure such as the Hosmer-Lemeshow test to assess how well the predicted probabilities match the observed outcomes should be performed.

Action: Please calculate and report the AUC (with 95% CI) and a goodness-of-fit metric for the final multivariate model in the Results section. If the model demonstrates poor fit or discrimination, this must be transparently discussed as a limitation.

**Response :**Modified. Hosmer-Lemeshow test:χ^2^=10.67, df=8, *p*=0.221. AUC of the multivariable model (with 95% confidence interval)=0.686 (95% CI: 0.514-0.858; *p*=0.04). Regarding model performance, ROC curve analysis yielded an AUC of 0.686 (95% CI: 0.514–0.858, *p* = 0.04), indicating marginal but statistically significant discriminatory ability. The Hosmer-Lemeshow test (χ² = 10.67, df = 8, *p* = 0.221) showed no significant deviation between predicted probabilities and observed values, suggesting good model calibration. Taken together, the model demonstrates an acceptable level of both discriminatory ability and goodness-of-fit.

c. ELSO Certification: Explicitly state if the center holds official ELSO certification or only domestic accreditation.

**Response :**Modified. Our hospital is a nationally accredited ECMO Quality Control Center which holds official ELSO certification.

d. Nurse-to-Patient Ratio: Clarify the "1.9" value. Use standard ratio format (e.g., 1:2 or 2:1).

**Response :**Modified. Nurse-to-patient ratio : 2:1.

#2 Data Presentation & Tables

a. Missing Data in Tables: In the table regarding baseline characteristics, the row labeled "admission diagnosis/n" reports a p-value of 0.448, yet the cells for the specific groups are empty. Reporting a p-value without the corresponding descriptive statistics is scientifically invalid.

**Response :**Thank you for pointing out this critical oversight. We completely agree that reporting a *p*-value without the corresponding descriptive statistics is scientifically invalid. We have now revised Table 2 (Baseline Characteristics) accordingly. The missing descriptive data for the variable "Admission diagnosis" have been added. For each diagnostic subgroup, we now present the frequency (n) and percentage (%) for both the infected and non-infected groups, alongside the *p*-value. This correction ensures that the statistical result is presented with the necessary context for proper interpretation.

b. Missing Captions and Definitions (Table 3): Table 3 appears to be raw output pasted directly into the document. It lacks a descriptive Title/Caption and Footnotes. Per PLOS ONE guidelines, tables must be self-explanatory. You must define all abbreviations (B, S.E., Wald, etc.) in the footnotes.

**Response :**Modified. Abbreviations: B, regression coefficient; S.E., standard error; Wald, Wald test; OR, odds ratio; CI, confidence interval.

b. Uninterpretable Table Formatting: The tables depicting microorganisms lack descriptive headers for the data columns (e.g., describing what the figures "14" and "3" represent). Remove all vertical gridlines and ensure every column has a clear header.

**Response :**Modified.Deleted.

#3 Editorial Integrity

a. Inappropriate Narrative Voice: The paragraph discussing the EPV and Confidence Intervals contains text copied directly from reviewer correspondence ("I must highlight that...") and defensive phrasing ("results... are usually reliable").

Action: Delete that paragraph and replace with: "Although the EPV ratio (>5) justifies the use of standard logistic regression, it is important to acknowledge that the 95% Confidence Interval for preoperative endotracheal intubation (OR=4.852, 95% CI: 1.174–20.059) remains wide. This width suggests a degree of estimation uncertainty, likely attributable to the limited number of events within this specific subgroup, and warrants a cautious interpretation of the magnitude of the risk."

**Response :**Modified.

b. Removal of Meta-Text: In the Methods, remove "In accordance with the request...". State facts directly (e.g., "Data access commenced on...").

**Response :**Modified. Data acess commenced on 1st June 2024.

c. Abstract/Methods Redundancy: Remove the disjointed sentence "This was a retrospective cohort study" from the middle of the Methods. Integrate it into the opening sentence.

**Response :**Removed.

#4 Scientific Consistency

a. Hypothetical Methods: Remove the sentence "If the EPV... is less than 5, Firth logistic regression... should be adopted". Since EPV was >5, this procedure was not performed and should not be listed.

**Response :**Removed.

b. Missing Statistical Test: The sentence "compared using either chi-square test , as appropriate" is incomplete. Correct to: "...compared using either the Chi-square test or Fisher's exact test, as appropriate."

**Response :**Modified. Categorical variables were expressed as frequencies (percentages) and compared using either chi-square test or Fisher's exact test,

#5 Formatting & Proofreading

a. Typographical Errors:

"VS" formatting: Change "27.7%VS7.9%" to "27.7% vs. 7.9%".

Spacing: Fix missing spaces after periods (e.g., "support.Our", "ICU.All").

Punctuation: Remove the colon in "According to relevant literature:".

Double Punctuation: Remove double periods (e.g., "[4]..").

**Response :**Modified.

b. Non-Standard Symbols: Remove circled numbers (①, ②) and use standard lists.

**Response :**Modified.

c. Syntax: Fix the dangling phrase starting with "Retrospective cohort study,the ECMO-related...".

**Response :**Modified. The dangling phrase has been removed.
