# Supplementary material for: Risk factors for healthcare-associated infections in children undergoing ECMO after cardiac surgery for congenital heart disease: A retrospective study
Source: PLoS One. 2026 May 11;21(5):e0348220. doi: 10.1371/journal.pone.0348220 (PMC13160298; doi:10.1371/journal.pone.0348220)
Supplement: Attachment — Submitted filename: Response_to_Reviewers_auresp_5.docx [file pone.0348220.s007.docx]

**Response to Reviewer #5:** Thank you very much for your constructive feedback. We have carefully addressed your valuable suggestions as follows:

1. Interpretation of Model Discrimination (AUC):

I commend the addition of the model performance metrics. The calibration of the model appears adequate (Hosmer-Lemeshow p=0.221). However, an interpretative adjustment regarding the discrimination metric (AUC) is required.

The reported AUC is 0.686 (95% CI: 0.514–0.858). In standard biostatistical practice, an AUC between 0.6 and 0.7 is generally classified as "poor" rather than "acceptable". Furthermore, the lower bound of the 95% CI (0.514) approaches 0.50 (random chance). Concluding that the model demonstrates an "acceptable level of both discriminatory ability and goodness-of-fit" overstates its predictive robustness.

Action Required (Results): Please retain the phrase "marginal but statistically significant discriminatory ability," but remove the concluding claim that the discrimination is "acceptable."

Action Required (Limitations): Please explicitly integrate this marginal discrimination into the Limitations section. Add a statement acknowledging that because the AUC is marginal and its lower confidence bound approaches 0.5, the predictive power of the multivariable model is limited, requiring a cautious clinical application of the reported Odds Ratios.

**Response :**Thank you for your insightful comments regarding the interpretation of our model's discrimination. We agree with your assessment and have revised the manuscript accordingly.

We acknowledge that an AUC of 0.686 (95% CI: 0.514–0.858) falls within the "poor" range per standard biostatistical practice, and that the lower confidence interval approaching 0.5 indicates limited predictive certainty.

****In the Results section:**** We have removed the characterization of the discrimination as "acceptable" and have retained the more precise phrasing describing it as having "marginal but statistically significant discriminatory ability."

****In the Limitations section:**** We have added a explicit statement acknowledging that the model's predictive power is limited due to the marginal AUC and its wide confidence interval. We emphasize that this necessitates cautious interpretation and application of the reported Odds Ratios in a clinical setting.

2. Inadequate Narrative and Missing Methodological Context for Statistical Tests:

The reporting of the multivariate analysis in the Results section reads like raw statistical software output rather than a cohesive scientific narrative. Appending isolated data fragments (e.g., "Hosmer-Lemeshow test: chi^2=10.67, df=8, p=0.221") without proper sentence structure is poor scientific writing. Furthermore, the methodology behind these specific tests must be formally introduced in the Methods section before the results are presented.

Action Required (Methods): Please update the Statistical Analysis section to explicitly state your methodology for evaluating the model (e.g., "Model calibration was assessed using the Hosmer-Lemeshow goodness-of-fit test, and discrimination was evaluated using the Area Under the Receiver Operating Characteristic curve (AUC).")

Action Required (Results): Please rewrite the isolated data fragments into proper, grammatically correct sentences that provide narrative context.

**Response :**We have made the following revisions:

**In the Methods section:** Model performance was evaluated using two metrics. First, model calibration—the agreement between observed and predicted outcomes—was assessed using the Hosmer-Lemeshow goodness-of-fit test, where a *p＞*0.05 indicates adequate calibration. Second, model discrimination—the ability of the model to distinguish between patients with and without the outcome—was evaluated by calculating the Area Under the Receiver Operating Characteristic curve (AUC).

**In the Results section:**  The multivariable logistic regression model demonstrated adequate calibration. The Hosmer-Lemeshow goodness-of-fit test yielded a non-significant result (χ²=10.67, df = 8, *p*= 0.221), indicating no significant deviation between observed and predicted event rates. Regarding model discrimination, the area under the receiver operating characteristic curve (AUC) was 0.686 (95% CI: 0.514-0.858), suggesting marginal but statistically significant discriminatory ability(Fig 2).

3. Inappropriate Prescriptive Language in Statistical Analysis:

In the Methods section, the sentence "Wherever expected cell frequencies are <5 in more than 20% of cells, the p-values must be recalculated using Fisher’s Exact Test" is stylistically inappropriate. The Methods section must serve as a descriptive record of the analysis actually performed (written in the past tense), rather than prescribing rules or pasting direct instructions from reviewers ("must be recalculated").

Action Required: Please revise this sentence to reflect standard, objective scientific writing (e.g., "Fisher’s exact test was used to compare categorical variables when expected cell frequencies were less than 5 in more than 20% of the cells.").

**Response :**We have revised the sentence accordingly to reflect standard, objective scientific writing in the past tense. Fisher’s exact test was used to compare categorical variables when expected cell frequencies were less than 5 in more than 20% of the cells.

4. Inappropriate Narrative Voice and Residual Rebuttal Text:

Similar to the issue above, the manuscript contains sentences written in the first-person singular that appear to be copied directly from rebuttal correspondence. Specifically, the phrase "I explicitly state that prior antimicrobial therapy might have suppressed bacterial growth" is highly inappropriate for a multi-authored scientific manuscript and breaks the objective tone of the paper.

Action Required: Please review the entire manuscript to remove any first-person singular pronouns ("I") and defensive/rebuttal phrasing. Revise this specific sentence to an objective scientific statement, such as: "Furthermore, prior antimicrobial therapy might have suppressed bacterial growth."

**Response : Modified.**

5. Residual Meta-Text in the Abstract:

The Abstract currently contains administrative details regarding the review process. The Abstract must serve as a standalone summary of the scientific work.

Action Required: Please completely delete the following sentences from the Abstract: "In accordance with the request for methodological transparency, we report that access to the dataset for the specific purposes of this study commenced on 1st June 2024. Formal data analysis specific to the research questions outlined herein began on 1st December 2024 and concluded on 1st March 2025. Statistical analysis was performed on the relevant factors."

**Response :** We have completely removed the specified sentences from the Abstract, as requested.

6. Phrasing and Typographical Errors in the Methods Timeline:

In the main text (Methods section), the newly added sentences detailing the timeline of data access contain a typographical error and overly bureaucratic phrasing.

Action Required: Please correct the typo ("Data acess") and simplify the syntax. I suggest replacing the current sentences with standard phrasing: "Data access began on June 1, 2024. Data analysis was conducted from December 1, 2024, to March 1, 2025."

**Response :**We have revised the sentences as per your suggestion to ensure clarity and professionalism.

7. Severe Readability and Formatting Issues (Text):

Despite previous requests to fix formatting and spacing, the manuscript suffers from a severe lack of readability. The text is poorly structured and riddled with non-standard punctuation.

Inclusion/Exclusion Criteria and Definitions of Infections: These sections are compressed, retain missing spaces ("telephone.Inclusion"), use non-standard punctuation ("1、"), and inexplicably include text left in red font (unaccepted track-changes).

Action Required: A meticulous, line-by-line proofreading of the entire manuscript is mandatory. Remove all non-standard symbols, ensure proper spacing after punctuation marks, use standard line breaks or bullet points for lists, and remove all residual track-change formatting (red text).

**Response :Modified.**

Inclusion Criteria:

1. Age < 18 years;
2. ECMO support duration > 48 hours.

Exclusion Criteria:

1. Presence of healthcare-associated infections before ECMO initiation;
2. Patients who required ECMO support before congenital heart surgery.

8. Inadequate Table Formatting:

Despite previous attention drawn to table presentation, the current tables remain highly difficult to read. The variable name cells are vertically expanded, while the corresponding data values remain vertically center-aligned in their respective cells. This misalignment forces the reader to guess which value belongs to which variable across the row.

Action Required: Please reformat all tables to ensure clear, horizontal alignment (e.g., top-aligning the contents of the cells or strictly ensuring line-by-line correspondence) so that readers can easily and accurately track the data rows.

**Response :**We have completely reformatted all tables to ensure clear horizontal alignment. Specifically, we have implemented top-alignment for all cell contents to guarantee strict line-by-line correspondence between variable names and their associated data values. This revision ensures that readers can easily and accurately track each data row.
