# Supplementary material for: Risk factors for healthcare-associated infections in children undergoing ECMO after cardiac surgery for congenital heart disease: A retrospective study
Source: PLoS One. 2026 May 11;21(5):e0348220. doi: 10.1371/journal.pone.0348220 (PMC13160298; doi:10.1371/journal.pone.0348220)
Supplement: Attachment — Submitted filename: Response_to_Reviewers_auresp_6.docx [file pone.0348220.s008.docx]

**Response to Reviewer Comments：**we appreciate the reviewers’ time and expertise in helping us improve this work.

****1. Interpretation of the multivariable model and causal language****

**The current manuscript continues to describe preoperative endotracheal intubation as an “independent risk factor.”
Given the small sample size, wide confidence intervals (OR 4.852, 95% CI 1.174–20.059), and marginal discrimination (AUC 0.686, 95% CI 0.514–0.858), this wording is too strong.**

**Please revise throughout the manuscript to use more appropriate language, such as:**

**“associated with increased risk”**

**“associated with the occurrence of HAI”**

**Causal implications should be avoided.**

**Response :**Thank you for your insightful comment regarding the interpretation of our multivariable model and the use of causal language. We completely agree with your assessment that given the relatively small sample size, the wide confidence intervals (especially for the variable of preoperative endotracheal intubation), and the modest discriminative ability of the model (AUC 0.686), the term "independent risk factor" was overly definitive and potentially misleading.

In response to your suggestion, we have carefully revised the manuscript throughout to temper our language. We have replaced all instances of causal or deterministic phrasing with more appropriate epidemiological terminology that emphasizes association rather than causation.

Specific changes include:

Throughout the manuscript: The term "independent risk factor" has been replaced with "was significantly associated with the occurence of ECMO-related HAI".

In the Results section: We now present the finding as: "preoperative endotracheal intubation remained significantly associated with the occurrence of HAI (OR 4.852, 95% CI 1.174–20.059, *P* < 0.05)."

In the Discussion section: We have added a note of caution regarding the instability of the estimate due to the wide confidence interval and the modest AUC, suggesting that these findings warrant validation in larger studies.

**2 : Confounding by severity (major methodological concern)**

**Preoperative intubation is very likely a marker of underlying clinical severity rather than a direct causal factor. This introduces a substantial risk of confounding by indication.**

**This issue is currently underdeveloped in the Discussion.**

**Please:**

**Explicitly acknowledge this limitation**

**Expand the discussion on how disease severity may have influenced both exposure (intubation) and outcome (infection)**

**Clarify that the observed association may not reflect a direct causal relationship.**

**Response :**We agree that preoperative intubation is likely a marker of underlying clinical severity rather than a direct causal factor. We have substantially revised the Limitations section to: (1) explicitly acknowledge confounding by indication; (2) expand discussion of how disease severity may influence both exposure and outcome; and (3) clarify that the observed association does not necessarily reflect a direct causal relationship.

**3:Model robustness and statistical limitations**

**Although you have improved the reporting of model performance, the limitations are still not sufficiently emphasized.**

**Please strengthen the Limitations section by clearly stating:**

**The small sample size and low number of events**

**The borderline events-per-variable ratio (risk of overfitting)**

**The wide confidence intervals indicating imprecision**

**The limited discriminatory performance (AUC close to 0.5 at the lower bound)**

**The model should be presented as exploratory and hypothesis-generating, not predictive.**

**Response :**We have strengthened the Limitations section to clearly state the small sample size, low number of events, borderline events-per-variable ratio, wide confidence intervals indicating imprecision, and limited discriminatory performance (AUC with lower bound near 0.5). We now explicitly characterize our findings as exploratory and hypothesis-generating.

**4: Definition and potential misclassification of infections**

**The study includes only microbiologically confirmed infections, while clinically suspected but culture-negative cases were excluded despite receiving treatment.**

**This approach introduces potential selection and misclassification bias, and may underestimate the true incidence of infection.**

**Additionally, the finding that 100% of infections were respiratory in origin warrants further discussion.**

**Please:**

**Clarify this methodological choice more explicitly**

**Discuss its implications and limitations**

**Address the potential bias introduced by culture-based definitions**

**Response :**We have clarified in the Methods section that only microbiologically confirmed infections were included, and in the Limitations section we discuss the potential for selection and misclassification bias, underestimation of true infection incidence, and the implications of relying on culture-based definitions. We have also added discussion regarding the predominance of respiratory infections in our cohort.

**5 : Interpretation of ICU length of stay**

**The manuscript discusses ICU length of stay as a variable associated with infection; however, this variable is likely influenced by the occurrence of infection itself (reverse causation).**

**Please:**

**Clarify the temporal relationship**

**Avoid implying that ICU stay is a causal factor for infection in this context**

**Revise the discussion accordingly**

**Response :**We acknowledge the potential for reverse causation between ICU length of stay and infection. We have revised the Discussion to clarify the temporal limitations and avoid implying that ICU stay is a causal factor for infection.

**6 : Overinterpretation and clinical recommendations**

**The Discussion includes extended recommendations (e.g., airway management protocols, VAP prevention strategies) that are not directly supported by the study data.**

**Please revise this section to:**

**Focus on findings supported by your results**

**Avoid guideline-style or prescriptive recommendations not derived from your analysis**

**Response :**We have deleted the relevant extended recommendations.

**7：Minor Points**

**The manuscript still contains minor issues with language, punctuation, and formatting (including tables and spacing). A careful, line-by-line proofreading is required.**

**Ensure consistency in terminology (e.g., HAI, ECMO-related infection).**

**Tables should be further improved to enhance readability and alignment.**

**Response :**We thank the reviewer for the careful reading and constructive suggestions. We have conducted a thorough line-by-line proofreading of the entire manuscript to address issues related to language, punctuation, and formatting. Terminology has been standardized throughout the text; "healthcare-associated infection (HAI)" and "ECMO-related HAI" are now used consistently. In addition, all tables have been reformatted to improve readability and alignment. We believe these revisions have substantially enhanced the overall quality and clarity of the manuscript.
